# Barriers to and enablers of prophylactic compression use by people at risk of venous leg ulcer recurrence: a qualitative study

**DOI:** 10.1136/bmjopen-2025-111730

**Published:** 2026-02-10

**Authors:** Abeer Muflih Alkahtani, Jo Dumville, Lily Mott, Chris Armitage

**Affiliations:** 1 The University of Manchester, Manchester, UK; 2 Manchester Centre for Health Psychology, University of Manchester, Manchester, UK

**Keywords:** WOUND MANAGEMENT, Patient-Centered Care, Behavior

## Abstract

**Abstract:**

**Background:**

Venous leg ulcers (VLUs) are complex, chronic wounds that often recur after healing. The ongoing use of compression hosiery is the primary strategy to reduce the risk of VLU recurrence. However, adherence to this prophylactic treatment is low, undermining treatment effectiveness and placing a substantial burden on individuals with a history of VLUs and on healthcare systems. Understanding the factors influencing people’s adherence to compression hosiery for secondary VLU prevention is essential to support approaches to promote uptake.

**Objectives:**

The study aimed to (1) draw on the Capabilities, Opportunities and Motivations of Behaviour (COM-B) model and the Theoretical Domains Framework (TDF) to explore factors influencing individuals’ use of prophylactic compression hosiery for the secondary prevention of VLUs and (2) use the behaviour change wheel to identify intervention strategies to support the ongoing use of prophylactic compression hosiery by individuals after VLU healing.

**Design:**

A descriptive, interpretive qualitative study involving individuals with a history of healed VLUs. Semistructured interviews were conducted with people who had experienced healed VLUs. The interviews were guided by the COM-B model. Framework analysis was conducted using deductive coding informed by the TDF and inductive coding to capture emerging themes linked to barriers to and enablers of the target behaviour (ongoing compression use). Data management was aided by NVivo software, and coding was conducted by two researchers.

**Settings:**

Interventions were conducted in person, by telephone or online, based on participants’ preferences, at community leg clubs or in their homes, from April 2024 to January 2025.

**Participants:**

Participants with experience of healed VLUs were recruited from three National Health Service (NHS) trusts and community leg clubs in the North of England.

**Results:**

A total of 15 participants were interviewed, comprising 4 males and 11 females aged between 49 and 89 years. Our analysis identified six factors that may influence individuals’ use of prophylactic compression hosiery following VLU healing: knowledge, skills, environmental context and resources, emotion, social influences and beliefs about consequences. Deficits in knowledge, skills and resources, such as limited availability of prophylactic compression sizes, delays in prophylactic compression delivery and limited access to NHS services after healing, were primary barriers to people’s use of compression hosiery in this context. Conversely, positive beliefs about the benefits of ongoing use of prophylactic compression hosiery were a strong enabler. Emotion and social influences were identified as both barriers and enablers: fear of recurrence and social support encouraged adherence, while stigma and negative feelings hindered it. We identified six intervention functions (education, training, persuasion, environmental restructuring, modelling and enablement) and eight linked behaviour change techniques that could be explored further to support people’s ongoing use of prophylactic compression therapy. These techniques include providing information about antecedents, discussing health and emotional consequences, instruction, demonstration, rehearsal, social support, framing/reframing and vicarious reinforcement.

**Conclusions:**

The identified intervention functions and behaviour change techniques provide theoretically informed insights for designing interventions to support sustained use of prophylactic compression hosiery following VLU healing. Key barriers to address include addressing gaps in individuals’ knowledge about prophylactic compression therapy, prioritising posthealing VLU services, ensuring timely access to appropriately fitted compression and enhancing social support networks.

**STRENGTHS AND LIMITATIONS OF THIS STUDY**

- This research provides a novel, theory-informed perspective on the factors that promote long-term adherence to prophylactic compression hosiery after the healing of (venous leg ulcers, VLUs).
- We recruited participants from two different care settings: NHS Trusts and Leg Clubs. This approach enabled us to consider behavioural determinants from individuals receiving different types of NHS care.
- The study suggests potential theoretically informed, behaviour change interventions for supporting ongoing compression hosiery use for people with a history of VLUs, using behaviour change theory.
- Despite expanding our recruitment channels, our sample remained predominantly White British. This limits our ability to examine how cultural norms, ethnic backgrounds and health beliefs might influence experiences with prophylactic compression therapy and VLUs prevention.

## Introduction

 Venous leg ulcers (VLUs) are chronic open wounds on the lower limb, between the knee and the ankle, caused by impaired venous return and resulting venous hypertension.^1 2^ A systematic review and meta-analysis estimated a global VLU prevalence of 0.32% (95% CI 0.129% to 0.595%).^3^ The point prevalence of VLUs in the UK population has been estimated at approximately 3.2 per 10 000.^4 5^ VLUs significantly burden affected individuals, causing persistent pain, limited mobility and emotional distress, which restrict daily activities and substantially diminish health-related quality of life.^6^

Compression therapy is the gold standard treatment for healing VLUs, with various types and strengths available (see online supplemental file A). Estimated VLU healing rates vary, with 42.2% of ulcers estimated to be healed within 3 months in a Canadian study^7^ and up to 70% of ulcers reportedly healed by 6 months in UK trials of different compression therapies.^8 9^ Endovenous ablation surgery combined with compression therapy has been shown to improve healing outcomes,^10^ although it is not yet widely adopted in UK practice^11 12^ and may not be suitable for all people with VLUs.^13^ Despite initial treatment success, the underlying venous pathology typically persists, making VLU recurrence a constant concern, affecting up to 70% of patients.^14^

The effectiveness of prophylactic compression hosiery (stockings) in preventing VLU recurrence is well-established.^15^ Its success, however, hinges on a specific target behaviour outlined by national and international guidelines^15^,^18^: the ongoing use of compression hosiery at the strongest level tolerated by the individuals (ideally ≥40 mm Hg at the ankle). Despite this evidence-based recommendation, posthealing adherence to the therapy is low.

Estimates of individual adherence rates to prophylactic compression therapy vary, with published figures ranging from 12% to 52% of those using prophylactic compression therapy as recommended.^19^ Suboptimal adherence likely contributes to persistently high VLU recurrence rates, imposing a burden on people’s health-related quality of life and healthcare systems in general.^20^,^23^

While prior research has identified individual-related factors influencing the use of compression therapy in the treatment of active VLUs,^24^,^26^ less attention has been given to the factors affecting the ongoing use of prophylactic compression after healing. This distinction is important, as compression therapy for ulcer healing is often applied by health professionals, typically by NHS nurses in the UK, whereas posthealing maintenance requires individuals to self-apply compression hosiery. This shift in responsibility may impose significant physical demands, especially with higher-strength compression hosiery, as it is often tight and can be particularly difficult for individuals to apply.

A greater depth of insight into factors that influence individuals’ perceptions and experiences of compression as a preventative treatment is needed to identify strategies that could impact people’s behaviours to support adherence. Unpacking this complex behaviour requires a multidimensional theoretical approach to examine the interplay of practical, psychological and perceptual factors.

Our study employs behaviour change theory, specifically the Capabilities, Opportunities, Motivations model of Behaviour (COM-B) and the associated Theoretical Domains Framework (TDF). The COM-B model posits that behaviour is determined by an individual’s capability (physical and psychological), opportunity (physical and social) and motivation (reflective and automatic).^27^ COM-B is central to the behaviour change wheel for developing interventions.^27 28^ The behaviour change wheel provides a systematic approach to designing interventions. It identifies intervention functions, policy categories and specific behaviour change techniques that should be employed. Emphasising behaviour change techniques is crucial, as an increasing amount of evidence indicates a direct link between specific techniques and user engagement in health interventions.^29 30^ Definitions of each component can be found in online supplemental file B.

The TDF further elaborates on these three core components into 14 specific domains, enabling a more detailed analysis of behavioural drivers.^28 31 32^ This multidimensional approach provides a robust foundation for identifying specific barriers and enablers, ultimately guiding the development of potential interventions that are not only clinically effective but also individual-sustainable in a real-world context.

## Aims

The present study aims to (1) explore factors influencing individuals’ use of prophylactic compression hosiery for the secondary prevention of VLUs, drawing on the COM-B and the TDF and (2) identify intervention strategies to support the ongoing use of prophylactic compression hosiery by individuals after VLU healing, guided by the behaviour change wheel.

## Methods

### Design and sampling

A qualitative study was conducted using semistructured interviews. We used criterion purposive sampling to recruit individuals who met predefined eligibility criteria, being aged 18 years or older, able to provide informed consent, proficient in written and spoken English, and having a history of one or more healed VLUs.^33^ To maximise participation and accommodate participant preferences, we offered interviews in multiple formats, including face-to-face, telephone and online via Zoom.^34^,^36^

### Data collection

Recruitment and data collection were conducted between April 2024 and January 2025, across two settings: community-based Leg Clubs and two NHS Trusts in the North of England.

In the leg club settings, lead nurses were contacted by email with an overview of the study, and site visits were arranged. During these visits, a member of the research team introduced the study, answered questions and provided participant information sheets to those interested. Interview mode (face-to-face, telephone or online) and timing were discussed and arranged on-site or via follow-up contact.

Within the NHS Trusts, recruitment was facilitated by a clinical lead, who identified eligible participants and, with their consent, shared contact details with the researcher, who then contacted them directly to discuss the study and arrange interviews. To support participation and acknowledge participants’ time and contribution, those who completed the interviews were offered a £20 shopping voucher as a token of appreciation.^37 38^ All individuals who were invited agreed to participate, and no participants withdrew after consent.

Interviews were conducted using an interview guide adapted from a validated tool developed by Keyworth *et al*,^39^ which was structured around the COM-B model.

To allow for a more detailed and granular exploration of the barriers and enablers within each COM-B component, we integrated specific prompts from the TDF.^31 32^ A complete list of all TDF definitions is provided in online supplemental file C. The full interview guide is presented in online supplemental file D.

Data collection and preliminary analysis occurred concurrently, and recruitment ceased when the research team determined that additional interviews were not yielding new insights, indicating data saturation—defined as the point at which no new themes or information emerged from the data.^40 41^ Interviews were conducted, audio-recorded and transcribed verbatim by the first author.

### Data analysis

All interview transcripts were anonymised and imported into NVivo software to facilitate data management.^42 43^ Returning transcripts to participants for review was not possible due to the geographical dispersion of the participants and the practical challenges of follow-up contact. In addition, many participants were dealing with health and mobility limitations, which made it difficult to engage with them afterward. Therefore, we omitted member checking while maintaining credibility through rigorous coding procedures.

The data were analysed using a framework approach, which integrates both deductive and inductive coding.^44^ The overall methodological orientation underpinning the study was a directed content analysis approach, which was appropriate as the initial interpretation was guided by established theory.^45^

The TDF provided the analytical structure, which enabled the identification, organisation and categorisation of participant responses within its relevant behavioural domains.

The analyses were conducted in two stages: first, a deductive coding process was carried out. In this stage, each transcript was read in full and coded against the relevant TDF domains,^31 32^ using established coding descriptions from the domain definitions.^46 47^

The importance of each domain was determined using two criteria: (1) the frequency and consistency with which a domain was identified across participants and (2) the presence of strong, explicit beliefs or experiences perceived by participants as barriers or enablers to ongoing prophylactic compression use. Domains that met both criteria were considered the most influential in shaping the behaviour.

Second, an inductive analysis was performed to generate explanatory themes from the most prominent TDF domains identified during the initial coding stage. Related codes were reviewed, compared and clustered into broader thematic categories capturing underlying mechanisms and contextual factors. This process involved iterative reading, constant comparison within and across cases, and integration of overlapping concepts to produce coherent, higher-order themes.^48^ Themes were supported by illustrative participant quotes and refined through multiple review cycles to ensure they remained grounded in participants’ narratives.

Participant validation of the final findings was not possible due to practical constraints. Nevertheless, trustworthiness was maintained through dual coding of transcripts, resolving discrepancies via discussion and a critical review by the multidisciplinary research team.

### Rigour and reflectivity

Methodological rigour was maintained through collaborative coding and reflexive team practice. Initial coding was carried out by two researchers to ensure consistency and interpretive alignment. Discrepancies were resolved through discussion, and the framework and themes were refined to enhance the trustworthiness of the analysis.^49^

The wider research team, including an applied health researcher with expertise in wound care and an expert in health psychology and behaviour change, brought diverse disciplinary and experiential perspectives to the analysis. These varied perspectives enabled a critical examination of underlying assumptions, challenged conventional interpretations and deepened analytic insights.^50^

Reflexive discussions in scheduled team meetings fostered integrative interpretation and contributed to refining and finalising the thematic structure, ultimately enhancing both analytical rigour and interpretive depth.^51 52^

There was no prior relationship between the interviewer and participants before recruitment. The clinical team approached the participants and provided an information sheet before inviting them to participate. Participants were informed that the interviewer was a PhD candidate without a clinical background conducting a qualitative study on behavioural factors related to compression hosiery use after healing. They were made aware of the study’s aims, interview formats and compensation before giving informed consent. The interviewer acknowledged having assumptions based on previous literature before data collection. These were alleviated through inductive coding, a second-coder review and discussions with the supervisory team, ensuring the analysis was grounded in participants’ experiences.

## Findings

### Participant characteristics

We included 15 participants, all of whom had previously healed VLUs and received wound care from NHS nurses in either community-based leg clubs or clinical settings. Among the participants, 11 (73.3%) reported experiencing one or more episodes of ulcer recurrence after their initial healing, while four participants (26.7%) did not experience any recurrence. Interviews ranged from 20 to 60 min (mean=32 min) and were conducted according to participant preference: online (Zoom, n=1), by telephone (n=5) and face-to-face (n=9). A summary of participants’ characteristics is presented in table 1. Participant-level self-reported co-occurring diagnoses are provided in online supplemental file E.

**Table 1:** Sample characteristics

Variable	n (%)
Gender	
Male	4 (27)
Female	11 (73)
Age	
40–49	2 (13)
50–59	2 (13)
60–69	1 (7)
70–79	5 (33)
80–89	5 (33)
Experience of recurrence venous leg ulcers	
Yes	11 (73.3)
No	4 (26.6)

**Figure 1:**
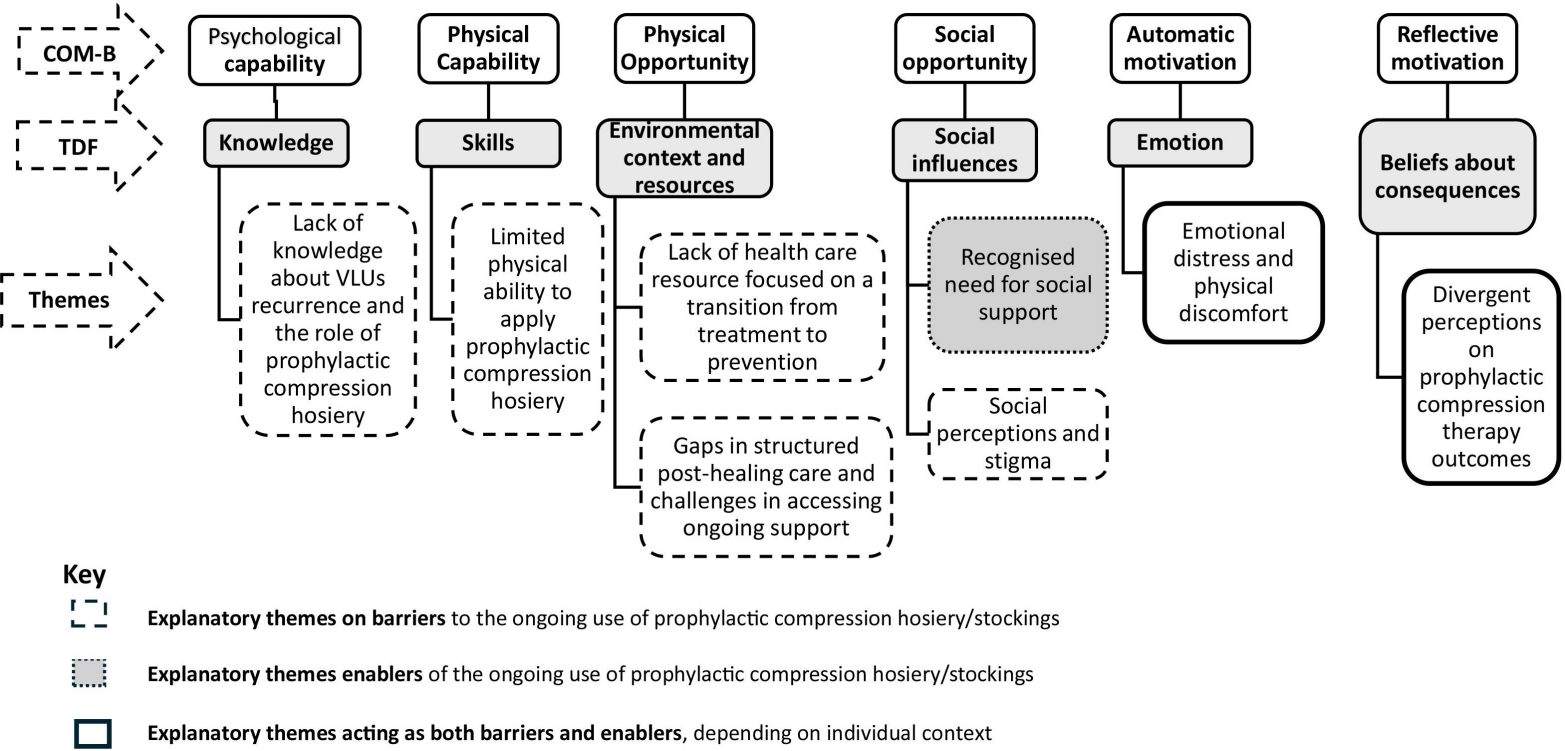
A summary of the factors influencing the ongoing use of prophylactic compression hosiery following healing. COM-B, Capabilities, Opportunities, Motivations model of Behaviour; TDF, Theoretical Domains Framework.

Our analysis of how behavioural determinants can influence the sustained use of prophylactic compression hosiery is summarised in figure 1, with additional details provided in the narrative. Table 2 presents a summary of findings, highlighting the identified barriers and enablers that affect the ongoing use of prophylactic compression hosiery among individuals with healed VLUs.

**Table 2:** Summary of key findings mapped to intervention functions and individual behaviour change techniques according to Michie *et al*^28^

COM-B component	TDF domain	Description of the COM-B and the TDF domain	Exemplar quote	Intervention function	Individual behaviour change technique	Candidate interventions
Psychological capability	Knowledge	Limited individual understanding of the causes of VLU, limited awareness of the necessity for lifelong compression to prevent recurrence and gaps in practical knowledge regarding types, aids and sizing hinders the continued use of prophylactic compression after healing.	*“No, I was never aware of anything like that…*” (Participant *003*) “I *haven’t a clue*…” (Participant 004).	Education Persuasion	Information about antecedents; Information about health consequences;	Explain VLU causes, recurrence and the important role of prophylactic compression hosiery and how it directly reduces or prevents recurrence. Intervention functions: (education; Behaviour Change Techniques: Information about antecedent).
Physical opportunity	Environmental context and resources	Long delivery delays of prophylactic compression and poor fit from standardised sizing limit accessibility and consistent use of prophylactic compression. The lack of clear transition from treatment to prevention, and limited NHS follow-up, hampered adherence to prophylactic compression.	*“I didn’t actually have it for overtwo months because it wasn’t available in stock*” (Participant 001). “…*they healed and I was just told be careful… that. Be vigilant…”* (Participant 014).	Environmental restructuring Enablement	Restructuring the physical environment Adding objects to environment Restructuring the social environment Social support practical	Enhance the ordering and delivery systems for prophylactic compression, expand size and fit options, and create a structured prevention phase after healing, with dedicated support for people. Intervention functions: (Environmental restructuring; Behaviour Change Techniques: Restructuring the physical environment and restructuring the social environment).
Social opportunity	Social influences	Family or community support enabled adherence, while lack of support led to dependency, isolation and reduced use. Negative perceptions, stigma and embarrassment—often reinforced by family scepticism or public visibility—undermined confidence and decreased long-term use of prophylactic compression.	*“…I can’t manage to get it on myself and… I don’t like to be reliant on people I want to do it myself….*” (Participant 015). *“…I hate it, you know…that social situation …I just don’t like how I feel when …you can see that my stockings there and I just don’t like it…”* (Participant 015).	Enablement Education	Social support (practical). Social support (emotional). Information about health consequences Information about emotional consequences	Social support (practical): Involving a family member, friend, or caregiver in the process to provide physical help (eg, with application/removal). Intervention functions: (Enablement; BCT: Social support practical). Helping individuals anticipate and plan how to handle potentially negative social situations or comments. Intervention functions: (Education; Behaviour Change Techniques:: Information about emotional consequences).
Automatic motivation	Emotion	Emotional distress and discomfort, such as heat and frustration, hindered the ongoing use of prophylactic compression, while the fear of recurrence strongly motivated adherence.	*“…I just don’t like tight things around my legs really…” (Participant 003*) *“to stop ulcers coming. ‘Because once you get those ulcers, you’ve had it for a long time” (Participant 005*).	Persuasion Modelling	Framing/reframing Information about health consequences	Transforming the individual’s view of prophylactic compression hosiery from a source of discomfort to a ‘shield’ or ‘protector’ against a far greater harm. Intervention functions: (Persuasion; Behaviour Change Techniques: Framing/reframing).
Reflective motivation	Beliefs about consequences	Recognising the health benefits of comfort, circulation and preventing recurrence encouraged sustained use of prophylactic compression. In contrast, scepticism about its effectiveness made the effort seem unnecessary.	“*Just the health benefits. I can clearly see that my legs are better for wearing the compression socks. My feet feel more comfortable …my circulation is improved …So just the health benefits themselves, enough to keep me wearing them” (Participant 009*.)	Persuasion Modelling	Pros and cons Vicarious reinforcement	Showing a sceptic that prophylactic compression hosiery has been effective for others with similar conditions can be very convincing. When they see a peer who was once a sceptic but has become a believer due to their positive results, it can be highly persuasive. Intervention functions:(Persuasion; Behaviour Change Techniques: Vicarious reinforcement).

COM-B, Capabilities, Opportunities, Motivations model of Behaviour; TDF, Theoretical Domains Framework; VLUs, venous leg ulcers.

### Domain 1: knowledge

#### Gaps in individuals’ knowledge (barrier)

Participants generally had limited insight about the cause of their previous VLUs and thus why ongoing compression hosiery use was promoted following healing. As a result of limited knowledge, some participants perceived prophylactic compression hosiery as unnecessary or irrelevant and struggled to initiate or sustain use. People noted that they prioritised other self-care strategies such as moisturisation, leg elevation and circulation exercises.

To be honest, I don’t know. I have no idea why … ulcer keeps happening. No idea…. (Participant 001).

Gaps in knowledge extended to practical aspects of compression hosiery use, with most participants being unaware of the different compression types, strengths or the availability of application aids, issues that contributed to uncertainty, disengagement and low sustained use. See table 2 for exemplar quote.

Of the 15 individuals interviewed, only four demonstrated a comprehensive understanding of prophylactic compression and its role in preventing recurrence. These participants typically had worked in healthcare, or health care-related roles, been involved in research, or had actively sought information themselves. In contrast, the majority relied on informal sources, most notably community leg clubs, which many viewed as key enablers of understanding and ongoing use.

I don’t know anything really about prevention or anything like that …. And I’ve got all that information from the …research team. And I’ve also checked it out online.… (Participant 003).

I did wear it until my ulcer healed and then I thought I don’t need to wear it anymore… since I’ve been going to the leg club really. I didn’t wear them before….’’ (Participant 005).

### Domain 2: skills

#### Limited physical ability to apply prophylactic compression hosiery (barrier)

Physical challenges and skill limitations in applying compression hosiery were reported as significant barriers, undermining individuals’ ability to initiate and sustain use.

Most participants reported difficulties related to coexisting health conditions such as arthritis (n=5), chronic back pain (n=4) and reduced mobility (n=7), placing significant physical constraints on their ability to apply and remove compression hosiery. This reduced capability increased frustration and dependency, often leading to diminished motivation for continued use, and in some cases, discontinuation.

Putting them on is the most difficult thing… you’ve got to sort of bend down and lift your leg up and… wriggle around… (Participant 002).

While a few participants viewed prophylactic compression hosiery as a skill that could be developed over time, this was generally limited to those without significant physical limitations.

Once you’ve done it a couple of times, you know how to put it on (Participant 004).

Challenges associated with the ongoing use of compression hosiery were particularly evident among those prescribed strong (Class 3) compression hosiery, which evidence suggests is the most effective for preventing VLU recurrence. Although applying this strength was uncommon, where maintained, it was supported by participants who had sufficient physical ability, prior experience with compression or a strong belief in its effectiveness. In contrast, many participants preferred reduced-strength compression (class 1 or class 2) or alternative methods, such as adjustable compression wraps, due to their ease of application and reported relative comfort.

I’m on Class 3. I have three stockings with a liner. That gives a total of about 40 millimetres of mercury….I wear the compression during the day. I keep the liner on at night which gives me a 10mm compression and then put the full one on.…’ (Participant 007).

I do have a stocking that I put on,… and number three, one pair I had to literally cutoff because they were really, really tight. They were horrible, …they were terrible…. So then they suggested that I tried these …leg wraps and they’re brilliant, absolutely fantastic (Participants 015).

See table 2 for more exemplar quotes.

### Domain 3: environmental context and resources

#### Lack of healthcare resource focused on a transition from treatment to prevention (barrier)

Limited availability and accessibility of prophylactic compression hosiery was described as a barrier to its continuous use. While a minority of individuals reported timely access to well-fitting prophylactic compression in various sizes, the majority encountered substantial delays, with some waiting up to 2 months for deliveries.

Ijust got it on a prescription but it took months—it was a good nine weeks for it to come in stock at my chemist. (Participant 001).

In addition, when hosiery was applied, people felt standardised sizing often failed to accommodate their specific leg measurements, leading to hosiery that could be too loose or tight. Participants then had to invest time, effort and repeated follow-ups to obtain appropriate hosiery. These logistical and supply challenges seemed to increase individuals’ frustration with the therapy when being used and reducing willingness to persist.

…the size that I got was probably too tight for the size of my leg… but the size bigger would have been too big… so I needed one in between…. if they’d have ordered the next size, it wouldn’t have been tight enough, which wouldn’t have helped me because I wouldn’t have had enough compression to help. (Participant 015).

#### Gaps in structured posthealing care and challenges in accessing ongoing support (barrier)

Participants reported that the transition from compression therapy as a treatment to a prophylactic treatment was an unclear process, which hindered their ongoing use of prophylactic compression hosiery. Across accounts, participants described how formal care pathways within the NHS often ceased immediately after ulcer healing and a final appointment where compression hosiery for prevention was provided. Unlike the treatment phase when the ulcer was present, participants saw prevention framed as the individual’s responsibility, but with little support.

…I’ve just basically been prescribed to wear class 2 compression stockings and if any case, if anything happens in the meantime, then I have to contact them immediately… (Participant 008).

Where available, community-based resources such as Leg Clubs were valued for providing continuity and social motivation. However, these services were not widely accessible, and in their absence, many participants described feeling isolated, unsupported and emotionally disengaged—reinforcing the perception that prophylactic compression was optional rather than essential. Without regular contact with NHS nurses, many participants reported discontinuing their use of prophylactic compression. See table 2 for additional exemplars.

When I get discharged…I never go back. I’ve never had any follow-ups, never… I think you should be able to have more access to see the nurse even when it’s healed…. For those that can’t travel, the nurses should come up, if you’re partially disabled, which I am, they should come to your home, not only if you’re housebound. (Participant 001).

### Domain 4: social influences

#### Recognised need for social support (enabler)

The availability of practical support from family and community services emerged as crucial in influencing participants’ adherence to prophylactic compression therapy.

For many participants, particularly those with comorbidities or functional limitations, the ability to apply prophylactic compression was entirely dependent on consistent support from spouses or other family members. When this informal support was reliable, it was a powerful enabler. It transformed a potential daily struggle into a manageable routine, allowing adherence to the prophylactic compression despite physical constraints.

So if my husband’s in from work … my husband will do it for me … But if my husband is sometimes finishes a bit later … or he’s not been …. then I have to wait for him to come in and … put it on (Participant 010).

In contrast, participants lacking close family support reported experiencing social isolation, resulting in a dependency on external services for practical assistance. For these individuals, again community-based initiatives, such as Leg Clubs, were viewed as essential enablers, providing skilled assistance, continuity of care and emotional reassurance. Attendance addressed practical barriers and improved participants’ understanding of prophylactic compression, thereby promoting its sustained use.

You know what I don’t have anybody here because I don’t have any family.… So you’ve just got to, … as I say that leg club been such a help to me… (Participant 005).

#### Social perceptions and stigma (barrier)

Negative social perceptions and experiences of stigma were described as important barriers to the ongoing use of prophylactic compression. Participants’ sustained use of prophylactic treatment was shaped not only by their own experiences but also by the reactions, attitudes and judgements of others. These social pressures directly introduce feelings of embarrassment, self-doubt and discomfort that, in some cases, lead to diminished long-term use. A few participants described how scepticism or dismissive remarks from family members or peers challenged their own beliefs about the value of prophylactic compression and could hinder its long-term use:

…my son keeps saying, why on earth do you wear these things? It’s ridiculous, they don’t seem to be doing anything. He’s the main antagonist… And that to a point I think is probably right… (Participant 011).

Some participants expressed increased self-consciousness about the visibility of prophylactic compression in public. While some felt comfortable wearing these garments socially, others experienced embarrassment and a desire to avoid unwanted attention, especially in formal or unfamiliar settings. These concerns negatively impacted their feelings towards wearing compression and sometimes led to not using them at all.

…I was always taught …you don’t stare at people … but people do don’t they? …. I just don’t like how I feel when I’m like you know you can see that my stockings there and I just don’t like it… (Participant 010).

…So it was embarrassing really because everyone would say well what have you done to your leg? And then you’d go into detail and I’d just say I’m going to have to disband it and that’s how I used to say you know… (Participant 014).

### Domain 5: emotion

#### Perceived emotional distress and physical discomfort (barrier and enabler)

Emotional responses to prophylactic compression were powerful, often conflicting, and drivers of behaviour. These feelings, ranging from frustration to fear—directly shaped participants’ motivation to initiate and sustain prophylactic therapy, acting as both a barrier and an enabler.

For some, negative emotional experiences, particularly during warmer months, were tied to physical discomfort, inconvenience or appearance-related concerns. In these cases, prophylactic compression was experienced as burdensome and emotionally draining, prompting temporary pauses or reductions in use. Such breaks were often framed as ways to restore comfort or regain a sense of normality, reflecting reduced emotional engagement and challenges to sustaining therapy in daily life:

…I suppose at one point in time it might get frustrating. There are times now when I would just like to leave them off for a while…, I might kind of leave them off for half a day or something once I’ve taken them off …it’s kind of a relaxing, that little feeling of normality for a while… (Participant 009).

…but sometimes in the summertime when it’s really, really hot, I haven’t worn them every day (Participant 012).

Conversely, for others, strong protective emotions—particularly fear of recurrence—acted as powerful enablers. Fear motivated adherence by reinforcing the perceived necessity of prophylactic compression and driving ongoing commitment to its use.

it’s just the thought of anything starting again it’s the fear of it coming back again … that’s enough to make me wear the compression stockings the fear it’s a fear, isn’t it? (Participant010).

### Domain 6: beliefs about consequences

#### Divergent perceptions on prophylactic compression therapy outcomes (barrier and enabler)

For a small number of participants who demonstrated a clear understanding of compression as a preventive measure, this knowledge, combined with direct experience of physical improvements and/or positive experiences of the preventive treatment, meant they viewed prophylactic compression as essential for maintaining ulcer healing. They linked its use to tangible benefits such as reduced discomfort and improved circulation.

Just the health benefits. I can clearly see that my legs are better for wearing the compression socks. …Obviously, I know, I understand that my circulation is improved by wearing them, so, Just the health benefits themselves, enough to keep me wearing them (Participant 009).

In contrast, many other participants expressed scepticism about the benefits of prophylactic compression after healing. This scepticism meant the daily effort, discomfort and lifestyle restrictions seemed excessive compared with any potential benefit.

…I’m very sceptical about it, I just don’t know whether it’s worth all the sort of effort of trying to get them on and off and not being able to have a shower when I want and all this sort of thing. I’m not convinced… (Participant 011).

#### Exemplar interventions using BCTs

Table 2 presents descriptions of domains along with exemplar quotes. Six of the nine intervention functions proposed by Michie *et al*^27^ were linked to six TDF domains: education, persuasion, training, environmental restructuring, enablement and modelling. 10 out of 16 behaviour change techniques groups were found relevant: shaping knowledge, goals and planning, association, antecedents, self-belief, comparison of behaviour, social support, natural consequences, repetition and substitution, and identity. Within these groupings, 15 unique behaviour change techniques were found relevant. For example, to target the domain of knowledge, an intervention could expand the care pathway to include posthealing care. This might involve sharing peer success stories about continued prophylactic compression (intervention functions: modelling; behaviour change technique: social comparison), emphasising the importance of ongoing prophylactic compression after healing (intervention functions: education; behaviour change technique: information about health consequences), and providing guidance and aids for applying and removing hosiery (intervention functions: training; behaviour change technique: instruction on how to perform the behaviour). Additionally, restructuring the care environment to ensure these practices are supported (intervention functions: environmental restructuring; behaviour change technique: restructuring the physical environment).

## Discussion

This study employed COMB and the TDF to explore the determinants of the sustained use of prophylactic compression hosiery following VLU healing.

This study makes two key contributions to the literature. First, we identified six distinct TDF domains, representing the behavioural determinants that directly influence ongoing use of prophylactic compression. Second, we applied the behaviour change wheel^27^ to map these findings to relevant intervention functions and behaviour change techniques that could be incorporated into a foundation for interventions aimed at improving adherence to prophylactic compression and thereby reducing or preventing recurrences.

Consistent with previous research,^53^,^57^ participants reported barriers such as pain, discomfort, body image concerns, practical limitations, scepticism around effectiveness and insufficient social support.

While inconsistent use of prophylactic compression is often framed as an individual-level issue, our data and previous work^12^ revealed that barriers also stem from structural limitations in the provision and accessibility of care post-healing. Some of these barriers might mirror other structural limitations such as a postcode lottery, where access to essential care is influenced by geographic location rather than clinical need.^58^

A key distinction in our findings is the difference between practical skills and belief-based capabilities. While Weller *et al* described people’s behaviour in terms of self-efficacy or confidence,^56^ participants in this study emphasised perceived skills (ie, individual’s ability or proficiency acquired through practice) rather than beliefs about capability (ie; individual’s beliefs about their ability to perform a task). Participants primarily described difficulties related to physical ability and practical skill, noting that challenges in applying compression hosiery arise not from a lack of motivation or confidence, but from the physical demands of the task, which requires dexterity, strength and flexibility. This suggests that, for a number of participants, barriers are more functional than psychological. Although application aids are available, these are often not offered or are inadequate in supporting effective use.

While our sample size was small, we did note that Leg Club attendees seemed to demonstrate greater understanding of compression’s preventative role, stronger beliefs in its benefits and more positive affective responses. These differences appeared to stem from access to ongoing nursing support, peer interaction and informal education. This aligns with previous evaluations of the Lindsay Leg Club model,^59^,^61^ showing that community-based care fosters long-term engagement with self-care therapies.

### Strengths and limitations

A key strength of this study is that it provides a theoretical foundation that may inform future research and intervention development to enhance adherence to prophylactic compression hosiery following healing. By exploring six key behavioural domains reported to influence people’s adherence, the study provides clear, theory-informed insights for potential intervention development.

Recruiting from two distinct settings (NHS Trusts and Leg Clubs) enabled comparisons across care models, offering a more comprehensive understanding of how service context affects the ongoing use of prophylactic compression following healing.

However, the study has some limitations. The small sample size (n=15) may limit the transferability of findings and may not represent the full range of experiences in the broader population.

The sample was predominantly White British, limiting exploration of how cultural norms, ethnic background or health beliefs may shape experiences with prophylactic compression. Another limitation of the study is that data on participants’ education and socioeconomic status were not collected. This omission makes it difficult to understand how these factors may affect the sustained use of prophylactic compression. While we were able to explore key barriers and enablers to ongoing use of prophylactic compression hosiery following healing, future research should include more diverse populations to explore how ethnicity, education and socioeconomic level and culture may shape experiences with post-healing care. A limitation of this study is the methodological variation caused by using different interview formats (face-to-face, online and telephone) to accommodate participant preferences. This may have led to slight differences in data richness and the interpretation of non-verbal cues.^62^

### Implications for practice

Our findings, analysed through a behavioural lens, provide initial insights that may inform the development of targeted interventions for improving the sustained use of prophylactic compression. By systematically mapping individual-reported challenges to specific behavioural determinants, this research suggests the direction for developing interventions. These findings indicate that a comprehensive intervention focusing on functional, social and motivational factors warrants further investigation.

Our findings echo previous studies^11 12^ showing that NHS services often prioritise the treatment of active ulcers, with limited or no structured posthealing support—potentially signalling to people that care ends once the wound has closed. Embedding post-healing care as an integral, routine stage, rather than as a secondary or optional phase, may enhance people’s adherence.

Mapping challenges to specific behavioural components could offer a structured basis for designing targeted strategies to improve the ongoing use of prophylactic compression hosiery and reduce recurrence, which has been shown in similar research, where integrated interventions targeting multiple behavioural domains were shown to be more effective.^63^,^65^

### Future research

Future research can build on these findings by codesigning and evaluating interventions that are based on them. To be effective, these interventions need to be practical, person-centred and feasible, which requires active collaboration between individuals receiving care and clinicians throughout the development process. Crucially, given the limitations of our sample, these interventions should be piloted across diverse care settings and with ethnically and culturally diverse populations to ensure their effectiveness and equity.

### Conclusions

Improving people’s understanding of the role of prophylactic compression hosiery after healing appears to be a key factor in boosting long-term use. Participants in this study highlighted the importance of ongoing support, both clinical and social, in helping them navigate challenges related to application, comfort and motivation. These insights point to important areas for future interventions, including enhanced follow-up care, better support for self-treatment and clearer communication about the preventive role of compression therapy. Addressing these areas may promote more sustainable use and ultimately could contribute to reduced recurrence of VLU.

## Supplementary material

10.1136/bmjopen-2025-111730 online supplemental file 1

10.1136/bmjopen-2025-111730 online supplemental file 2

10.1136/bmjopen-2025-111730 online supplemental file 3

10.1136/bmjopen-2025-111730 online supplemental file 4

10.1136/bmjopen-2025-111730 online supplemental file 5

## Data Availability

Data are available on reasonable request.
