# Supplementary material for: Barriers to and enablers of prophylactic compression use by people at risk of venous leg ulcer recurrence: a qualitative study
Source: BMJ Open. 2026 Feb 10;16(2):e111730. doi: 10.1136/bmjopen-2025-111730 (PMC12911738; doi:10.1136/bmjopen-2025-111730)
Supplement: online supplemental file 1 [file bmjopen-16-2-s001.docx]

**Supplementary file A**

Compression Strengths. Taken from , E. A. Nelson and S. E. Bell-Syer (2014), Health Quality (2019), and the Australian and Jull A (2009)

| **Compression class** | **British Standard (mmHg)*** | **European Classification (mmHg** | **Australia/New Zealand (mmHg)** | **North America (mmHg)** |
| --- | --- | --- | --- | --- |
| Class 1 (light) | 14–17 | 18–21 | Extra light (5) | Supports 15–20 |
| Class 2 (medium | 18–24 | 23–32 | Mild (18–24) | Class 1: 20–30 |
| Class 3 (high) | 25–35 | 34–46 | Moderate (20–40) | Class 2: 30–40 |
| Class 4 (very high) | Not available | 49–70 | Strong (40–60), Very strong (>60) | Class 3: 40–50, Class 4: >50 |
| Class 4 super | Not available | 60–90 | Not available | Not available |

**Note*:**

- **mmHg** refers to millimetres of mercury, a measure of pressure.
