# Supplementary material for: Barriers to and enablers of prophylactic compression use by people at risk of venous leg ulcer recurrence: a qualitative study
Source: BMJ Open. 2026 Feb 10;16(2):e111730. doi: 10.1136/bmjopen-2025-111730 (PMC12911738; doi:10.1136/bmjopen-2025-111730)
Supplement: online supplemental file 2 [file bmjopen-16-2-s002.docx]

**Supplementary file B**

Definitions of the COM-B components, taken and adapted from Keyworth et al. (2020).

**What is PHYSICAL capability?**

Having the physical skill, strength or stamina to engage in the activity concerned.

(e.g. I have sufficient physical stamina, I can overcome disability, I have sufficient physical skills).

**What is PSYCHOLOGICAL capability?**

Knowledge and/or psychological skills, strength or stamina to engage in the necessary thought processes, for the activity concerned. (e.g. having the knowledge, cognitive and interpersonal skills, having the ability to engage in appropriate memory, attention and decision-making processes).

**What is PHYSICAL opportunity?**

The environment provides the opportunity to engage in the activity concerned. (e.g. sufficient time, the necessary materials, reminders).

**What is SOCIAL opportunity?**

Interpersonal influences, social cues and cultural norms provide the opportunity to engage in the activity concerned (e.g., support from friends and family).

**What is motivation?**

Conscious planning and evaluations (beliefs about what is good and bad) (e.g. I have the desire to, I feel the need to).

**What is automatic motivation**

It involves doing something without thinking or having to consciously remember (e.g. ‘is something I do before I realise I’m doing it’).
