# Supplementary material for: Barriers to and enablers of prophylactic compression use by people at risk of venous leg ulcer recurrence: a qualitative study
Source: BMJ Open. 2026 Feb 10;16(2):e111730. doi: 10.1136/bmjopen-2025-111730 (PMC12911738; doi:10.1136/bmjopen-2025-111730)
Supplement: online supplemental file 3 [file bmjopen-16-2-s003.docx]

| *Theoretical Domains Framework: Domains and definitions according to Cane, O’Connor and Michie (2012).* | |
| --- | --- |
| **Domain** | **Definition** |
| Knowledge | An awareness of the existence of something. |
|  | |
| Skills | An ability or proficiency acquired through practice. |
| Social/professional role and identity | A coherent set of behaviours and displayed personal qualities of an individual in a social or work setting) |
|  |  |
| Beliefs about capabilities | Acceptance of the truth, reality or validity about an ability, talent or facility that a person can put to constructive use. |
|  |  |
| Optimism | The confidence that things will happen for the best or that desired goals will be attained. |
|  | |
| Beliefs about Consequences | Acceptance of the truth, reality, or validity about outcomes of a behaviour in a given situation. |
|  | |
| Reinforcement | Increasing the probability of a response by arranging a dependent relationship, or contingency, between the response and a given stimulus. |
|  | |
| Intentions | A conscious decision to perform a behaviour or a resolve to act in a certain way. |
|  | |
| Goals | Mental representations of outcomes or end states that an individual wants to achieve. |
|  | |
| Memory, attention and decision processes | The ability to retain information, focus selectively on aspects of the environment and choose between two or more alternatives. |
|  |  |
| Environmental context and resources | Any circumstance of a person’s situation or environment that discourages or encourages the development of skills and abilities, independence, social competence and adaptive behaviour. |
|  |  |
| Social influences | Those interpersonal processes that can cause individuals to change their thoughts, feelings, or behaviours. |
|  | |
| Emotion | A complex reaction pattern, involving experiential, behavioural, and physiological elements, by which the individual attempts to deal with a personally significant matter or event (A complex reaction pattern, involving experiential, behavioural, and physiological elements, by which the individual attempts to deal with a personally significant matter or event. |
|  | |
| Behavioural regulation | Anything aimed at managing or changing objectively observed or measured actions |
| . | |
