# Supplementary material for: Barriers to and enablers of prophylactic compression use by people at risk of venous leg ulcer recurrence: a qualitative study
Source: BMJ Open. 2026 Feb 10;16(2):e111730. doi: 10.1136/bmjopen-2025-111730 (PMC12911738; doi:10.1136/bmjopen-2025-111730)
Supplement: online supplemental file 4 [file bmjopen-16-2-s004.pdf]

Title: Barriers to and enablers of using prophylactic compression for people at risk of venous leg ulcers to prevent recurrence: A qualitative study.

Interview schedule-patients' interview.

I am Abeer Alkahtani, a PhD student at the University of Manchester and my supervisory team includes Professor Jo Dumville and Professor Chris Armitage from the University of Manchester. As part of my PhD project, we are aiming to understand: (1) why people with VLUs wear or stop wearing compression to prevent re-ulceration; and (2) patients' acceptance of wearing compression to prevent re-ulceration.

Interview Question on both activities	Prompts
I know something about venous leg ulcers, but I am not a nurse and have not experienced venous leg ulcers. Would you be able to talk to me through about venous leg ulcer you're experiencing/ have experienced, please?	➢ Why do you think ulcers might come back once they have healed?<br>➢ What sort of things do you do to try and stop venous leg ulcers coming back?<br>➢ What preventive treatments have you been offered by a nurse (compression such as compression stockings/wraps?<br>➢ ***Explore****: what kind of compression? how frequent? in which level (strength?)*<br>➢ Do you know why you have been offered compression after your venous leg ulcer has healed? If yes: can you tell me why you have been offered compression?<br>➢ Do you think that people with healed VLUs have an active role in preventing the re-ulceration? Depending on response:<br>➢ **If yes:** can you talk to me about your role in preventing ulcer from coming back? **If no:** why?<br>➢ Do you feel there are any gaps in what you know about recommended approaches to care? **If yes**: what the sources you rely on to know how to prevent re-ulceration?<br>➢ If **no**: why? How can this be improved?
Do you have the **PHYSICAL opportunity** to wear stockings or wraps to prevent recurrence of VLUs**?** **PHYSICAL opportunity means** The environment provides the opportunity **to engage in the activity concerned (wearing stockings, wraps)** (e.g. sufficient time, the necessary materials, reminders).	What situations make wearing compression easy /difficult?<br><br>When and how do you normally wear/remove compression?<br>- Depending on response: e.g. the availability of the size or aids needed to wear compression?

Do you have the **SOCIAL opportunity** to wear stockings or wraps to prevent recurrence of VLUs? **SOCIAL opportunity means** Interpersonal influences, social cues and cultural norms provide the opportunity to engage in the activity concerned.	- Do others influence whether you wear or not wear compression?<br>If yes, who and how?<br><br>Do you receive any support to continue wearing ongoing and the tightest compression stockings (or wraps)?<br><br>**If yes**: who and how?<br><br>Is there anything that encourage you to continue wearing compression?<br><br>**If yes**: what and how?
Are you motivated to wear stockings or wraps to prevent recurrence of VLUs? Motivation means conscious planning and evaluations (beliefs about what is good and bad) e.g. you have the desire to, you feel you need to.<br><br>How do you feel about wearing tightest stocking or wrap?	How does wearing compression stockings (or other approach) make you feel?<br><br>how tight the compression impact on how you feel?<br><br>What motivates you to *or prevents you from* wearing strongest (or tightest) compression?<br><br>*Depending on response: who and how do you think could help motivate you to wear compression?
Is wearing stockings / wraps to prevent re-ulceration is something that you do **automatical**ly? **Automatic motivation** involves doing something without thinking or having to consciously remember.(e.g. 'is something I do before I realise I'm doing it).	Is wearing compression a part of your daily routine?<br>**If yes:** Can you talk to me about your daily routine in wearing compression? When you normally wear it? How? When do you remove it?<br>How much effort does it take to wear compression (tightest stockings or wraps)?**If no**: why? What might help you to add it to your daily routine?
Are you **PHYSICALLY able** to wear stockings/wraps to prevent re-ulceration? **Physical capability means** Having the physical skill, strength or stamina to engage in the activity concerned. (e.g. I have sufficient physical stamina, I can overcome disability, I have sufficient physical skills)	What sort of skills do you need to apply the compression stockings (or other type of compression e.g. if using wraps)? Do you feel that you have these skills.<br>If no: what might help you develop them?
Are you **PSYCHOLOGICALLY able** to wear stockings/wraps to prevent re-ulceration?<br>**Psychological capability means** Knowledge and/or psychological skills, strength or stamina to engage in the necessary thought processes for the activity concerned. (e.g. having the knowledge, cognitive and interpersonal skills, having the ability to engage in appropriate memory, attention and decision making processes).	What can impact on your decisions to wear or not wear compression?<br><br>Is it easy to remember to wear compression?<br><br>Does anyone who isn't a nurse, for example a friend or family member support you in wearing compression?<br><br>Can you imagine wearing compression for the rest of your life? Would anything make this easier for you? If yes: what is it?

How fair is tightest stockings/wraps for people with healed VLUs?	Depending on response: Unfair*:* How and why?
How acceptable wearing tightest stockings/wraps to prevent reulceration?	Depending on response: if not acceptable: why?<br>➢ What could make it acceptable?
What do you think need to be improved that could encourage people with healed VLUs to wear ongoing compression, and ideally the strongest compression they can tolerate?	
Would like to add anything that I did not cover in my questions?	
