# Supplementary material for: Barriers to and enablers of prophylactic compression use by people at risk of venous leg ulcer recurrence: a qualitative study
Source: BMJ Open. 2026 Feb 10;16(2):e111730. doi: 10.1136/bmjopen-2025-111730 (PMC12911738; doi:10.1136/bmjopen-2025-111730)
Supplement: online supplemental file 5 [file bmjopen-16-2-s005.docx]

| **Participant ID** | **Long- term condition/s** |
| --- | --- |
| Participant 01 | Diabetes, heart failure and *chronic condition affecting mobility |
| Participant 02 | Diabetes, heart failure, stroke and chronic condition affecting mobility |
| Participant 03 | back pain |
| Participant 04 | Autoimmune conditions and eczema |
| Participant 05 | Rheumatoid arthritis and back pain |
| Participant 06 | None |
| Participant 07 | None |
| Participant 08 | Rheumatoid arthritis and Chronic condition affecting mobility |
| Participant 09 | Chronic back pain and chronic condition affecting mobility |
| Participant 010 | Chronic condition affecting mobility |
| Participant 011 | Diabetes and chronic condition affecting mobility |
| Participant 012 | Rheumatoid arthritis |
| Participant 013 | Rheumatoid arthritis and chronic condition affecting mobility |
| Participant 014 | Rheumatoid arthritis and back pain |
| Participant 015 | None |

**Supplementary E. Self-reported co-occurring diagnoses by participant**

* To ensure participant anonymity, specific procedures were omitted from reporting. Instead, conditions are described based on their functional impact.
