# Supplementary material for: Barriers to and enablers of prophylactic compression use by people at risk of venous leg ulcer recurrence: a qualitative study
Source: BMJ Open. 2026 Feb 10;16(2):e111730. doi: 10.1136/bmjopen-2025-111730 (PMC12911738; doi:10.1136/bmjopen-2025-111730)
Supplement: Supplementary file 6 [file bmjopen-2025-111730.reviewer_comments.pdf]

PEER REVIEW HISTORY

BMJ Open publishes all reviews undertaken for accepted manuscripts. Reviewers are asked to complete a checklist review form and are provided with free text boxes to elaborate on their assessment. These free text comments are reproduced below.

ARTICLE DETAILS

Title (Provisional)

Barriers to and enablers of prophylactic compression use by people at risk of venous leg ulcer recurrence: A qualitative study

Authors

Alkahtani, Abeer Muflih; Dumville, Jo; Mott, Lily; Armitage, Chris

---

VERSION 1 - REVIEW

**Reviewer**	**1**
**Name**	**Ogrin, Rajna**
**Affiliation**	**Bolton Clarke Support Centre, Bolton Clarke Research Institute**
**Date**	**31-Oct-2025**
**COI**	**None**

Thanks to the authors for undertaking this work. Wound care is an area in which COM-B and TDF can play significant roles, and I am pleased to have been able to review this work. Well done!

Overall comments
Overall, a well written, well thought out manuscript and project. Congratulations.

To improve this work, suggest:

- please use person centred language throughout (avoiding the term 'patient' and use individual, person, community member etc...
- Please follow reporting guidelines for qualitative studies to include all the important information. A good guideline is COREQ: Consolidated criteria for reporting qualitative research (COREQ): a 32-item checklist for interviews and focus groups | International Journal for Quality in Health Care | Oxford Academic

Specific comments

Abstract:

1. Suggest starting with a background/introduction to lead with the 'Why' of the work would be helpful;
2. Methods – How were the participants recruited? From where?
3. Results – please include details of the participants: number, age, gender.

Introduction:
It would be helpful to include some information about the impacts of VLUs on the lives of people living with them.

Methods:
Consider data saturation as a concept – a useful work to read is from the Braun and Clarke team: [To saturate or not to saturate? Questioning data saturation as a useful concept for thematic analysis and sample-size rationales: Qualitative Research in Sport, Exercise and Health: Vol 13 , No 2 - Get Access]

Highlighting from the COREQ checklist areas that are missing:
Methodological orientation and Theory
What methodological orientation was stated to underpin the study? e.g. grounded theory, discourse analysis, ethnography, phenomenology, content analysis

Personal Characteristics of research team
1. Interviewer/facilitator Which author/s conducted the interview or focus group?
2. Credentials What were the researcher's credentials? E.g. PhD, MD
3. Occupation What was their occupation at the time of the study?
4. Gender Was the researcher male or female?
5. Experience and training What experience or training did the researcher have?

Relationship with participants
6. Relationship established Was a relationship established prior to study commencement?
7. Participant knowledge of the
interviewer
What did the participants know about the researcher? e.g. personal goals, reasons for doing the research
8. Interviewer characteristics What characteristics were reported about the interviewer/facilitator? e.g. Bias, assumptions,
reasons and interests in the research topic
Participant selection
13. Non-participation How many people refused to participate or dropped out? Reasons?

Data collection
23. Transcripts returned Were transcripts returned to participants for comment and/or correction?

Data analysis
28. Participant checking Did participants provide feedback on the findings?

Results
The figure is great – well done.

Table 2 – suggest reframing 'poor patient understanding' to a less loaded term. 'Poor' implies it is the person at fault, as opposed to there being sub-optimal methods for enabling community members to learn about this area. That community members don't understand means we aren't conveying information in a form to enable this.

The age range was wide – with 10 of the 15 70 years and older – does this need mentioning? Is there any difference between younger and older participants?

Discussion
A great first couple of paragraphs, however there is limited discussion re: where this fits in the existing literature. A few things raised by participants related to lack of knowledge, yet they were receiving care – why is this lack of knowledge happening? Why is there vague transition from healing to prevention? What system level problems need to be further explored re: person-centred care delivery – ie. providing information in a form that people understand and can act on and having a clear transition process. What are the healthcare providers roles and responsibilities?

Perhaps include other studies in related fields that have done work with COM-B and TDF and progressed to co-design and implementation – and what needs to happen. It's not easy! A number of system issues need to also be considered.

Strengths and limitations

Re: participants – education level, socio-economic level can also be an issue – what else needs to be considered?

**Reviewer**	**2**
**Name**	**Garrison, Sheldon**
**Affiliation Institute**	**Advocate Aurora Health, Advocate Aurora Research**
**Date**	**06-Nov-2025**
**COI**	**None**

The topic is relevant and important. The study is interesting, and the analytical approach aids interpretation. If this manuscript is revised, it would be beneficial for the authors to focus on the strengths of the interviews rather than attempting to generalize the content herein.

1. Abstract Formatting (P1, L5): Revise according to the journal's formatting requirements. Adding Aims in this manuscript was unexpected.

2. Sample Size (n=15): The small sample raises concerns when addressing such a large patient population. Because treatment plans are often highly individualized, findings/conclusions in any form should not be generalized. While recruiting a larger sample would likely be impractical, the reported findings should be described qualitatively. For example, P1, L52 ("six potential factors potentially influencing...") to remove conclusiveness of your findings. This should be revised consistently throughout.

3. The use of multiple interview formats (e.g., Zooom, f2f) may have unintended effects on participant responses. This limitation should be clearly noted.

4. Use fewer abbreviations. The journal's broad audience will better understand findings presented with full terminology in most cases.

5. The COM-B framework terms (e.g., psychological capability, physical capability) in table 2 are nebulous and provide little insight into what is being assessed. Clarify these terms.

6. Avoid using “first study (ex. P2, L57) ” as this claim is difficult to substantiate and likely inaccurate.

7. All abbreviations or acronyms must be written in full on first use and kept consistent throughout. Current usage is repetitive and inconsistent.

8. Co-Occurring Diagnoses (P18, L31): Instead of listing general examples, provide specific co-occurring diagnoses for each individual to enhance relevance.

9. Many points in the Results section refer to data not cited in tables or figures, or at least no link to either. Ensure all references are properly referenced.

10. If data are available, list the specific therapies used to treat ulcers. For example, participants undergoing hyperbaric oxygen therapy may have very different insights compared to those who did not undergo that type of treatment. The current data (Domain 4) are insufficient for interpretation without this context.

11. Do not list individual researcher roles within the Methods and other sections. Summarize author roles collectively at the end of the manuscript.

12. The study strengths and implications for practice are significantly overstated. The study is inherently interesting without generalizing the impact into findings that are ready to be used by other investigators. Reduce claims regarding broader impact and ensure any statement is consistent with the level of your data.

13. Study Limitations: Expand this section to address issues noted in this review, including sample size, interview variability, lack of therapy-specific data, etc.

14. Remove the future research section. The current study is quality-improvement oriented and not designed to establish a solid basis for future research.

15. I have serious concerns about the level and frequency of grammatical errors throughout. Significant revision is required to address these.

VERSION 1 - AUTHOR RESPONSE

**Reviewer 1 comments**	**Author response and any edits made**<br>all the comments from the editor(s)' have been addressed and highlighted in Yellow
**Overall comments:**<br>To improve this work, suggest:<br>please use person centred language throughout (avoiding the term ‘patient’ and use individual, person, community member etc.	We appreciate this comment and have adopted person-centred language throughout, replacing *“patient”* with terms like *“individual”*, *“people ”*, or participants as suggested.

Please follow reporting guidelines for qualitative studies to include all the important information. A good guideline is COREQ: Consolidated criteria for reporting qualitative research (COREQ): a 32-item checklist for interviews and focus groups | International Journal for Quality in Health Care | Oxford Academic.	Thank you for this valuable suggestion. We have reviewed the manuscript according to the COREQ (Consolidated Criteria for Reporting Qualitative Research) guidelines. We have addressed and clarified the relevant reporting aspects throughout the manuscript, as outlined in our responses to the ***specific comments*** below.
**Review 1 specific comments**	All the comments from the editor(s)' have been addressed and highlighted in Yellow
**Abstract:**<br>Suggest starting with a **background/introduction** to lead with the 'Why' of the work would be helpful;<br>**Methods** – How were the participants recruited? From where?<br>**Results** – please include details of the participants: number, age, gender.<br><br>**Introduction**:<br>It would be helpful to include some information about the impacts of VLUs on the lives of people living with them.<br><br>**Methods**:<br>Consider data saturation as a concept – a useful work to read is from the Braun and Clarke team: To saturate or not to saturate? Questioning data saturation as a useful concept for thematic analysis and sample-size rationales: Qualitative Research in Sport, Exercise and Health: Vol 13 , No 2 - Get Access	Thank you for the comment. The suggested points have been addressed and incorporated into the sections accordingly:<br><br>-We have added a brief introduction to the abstract (page 1)<br><br>- We followed BMJ Open's guidance and added the relevant information about recruitment to the "Participants" section (Page 2).<br><br>-We included the number, ages, and gender of participants in the Results section of the abstract (Page 2).<br><br>We have added a brief information about the impacts of VLUs on health-related quality of life (page 4)<br><br>Data saturation has been considered and referred to in Data collection section (Page 8).
**Highlighting from the COREQ checklist areas that are missing:**<br>**Methodological orientation and Theory:** What methodological orientation was stated to underpin the	The methodological orientation has been added to Data Analysis section (page 9)

study? e.g. grounded theory, discourse analysis, ethnography, phenomenology, content analysis.<br>**Personal Characteristics of research team**<br>1. Interviewer/facilitator Which author/s conducted the interview or focus group?<br>2. Credentials What were the researcher's credentials? E.g. PhD, MD<br>3. Occupation What was their occupation at the time of the study?<br>4. Gender Was the researcher male or female? 5. Experience and training What experience or training did the researcher have?<br>**Relationship with participants**<br>6. Relationship established Was a relationship established prior to study commencement.<br>7. Participant knowledge of the interviewer What did the participants know about the researcher? e.g. personal goals, reasons for doing the research<br>8. Interviewer characteristics What characteristics were reported about the interviewer/facilitator? e.g. Bias, assumptions, reasons and interests in the research topic<br>**Participant selection**<br>13. Non-participation How many people refused to participate or dropped out? Reasons?<br>**Data collection**<br>23. **Transcripts returned**<br>Were transcripts returned to participants for comment and/or correction?<br>**Data analysis**<br>28**. Participant checking**<br>Did participants provide feedback on the findings?	The characteristics of the research team have been included in the Rigour and Reflexivity section (page 10).<br><br>Points from 6-8 have been addressed in the Rigour and Reflexivity section (page 11).<br><br>In Data Collection section, we have added :'' *All individuals who were invited agreed to participate, and no participants withdrew after consent.*'' (Page 8)<br><br>Points 23 and 28 were both clarified and added to the Data Analysis section (Page 9)
**Results**<br>Table 2 – suggest reframing 'poor patient understanding' to a less loaded term. 'Poor' implies it is the person at fault, as opposed to there being sub-optimal methods for enabling community members to learn about this area. That community members don't understand means we aren't conveying information in a form to enable this.	Thank you for highlighting this. The wording has been revised from *"poor*'' Poor has been replaced with *''limited''*. (Table 2).
The age range was wide – with 10 of the 15 70 years and older – does this need mentioning? Is there any difference between younger and older participants?	Thank you for comment. Due to the small sample size in this study, we were unable to examine differences between younger and older participants. Additionally, investigating age-related differences was not part of the study

	aims. We have clarified the age distribution in the manuscript to better describe the characteristics of our participants, but we have not included any comparative analyses**. No changes have been made to the transcript.**
**Strengths and limitations**<br>Re: participants – education level, socio-economic level can also be an issue – what else needs to be considered.	Thank you for highlighting this. The comment has been addressed and highlighted in Strengths and Limitations section (Page 28).
**Reviewer: 2**	**Author response and any edits made**<br>all the comments from the editor(s)' have been addressed and highlighted in Blue
Dr. Sheldon Garrison, Advocate Aurora Health<br>Comments to the Author:<br>The topic is relevant and important. The study is interesting, and the analytical approach aids interpretation. If this manuscript is revised, it would be beneficial for the authors to focus on the strengths of the interviews rather than attempting to generalize the content herein.	We sincerely thank the reviewer for their positive and encouraging comments of our study.
1- Abstract Formatting (P1, L5): Revise according to the journal's formatting requirements. Adding Aims in this manuscript was unexpected.	We have revised the abstract to align with the journal's formatting guidelines. However, we have included a brief background to address the first reviewer's comment and to provide context for the study.
2-Sample Size (n=15): The small sample raises concerns when addressing such a large patient population. Because treatment plans are often highly individualized, findings/conclusions in any form should not be generalized. While recruiting a larger sample would likely be impractical, the reported findings should be described qualitatively. For example, P1, L52 ("six potential factors potentially influencing...") to remove conclusiveness of your findings. This should be revised consistently throughout.	We appreciate the reviewer's insightful comment. We recognise the concern regarding the small sample size and the importance of avoiding overgeneralisation in qualitative research. Our aim was not to generalise the findings, but to present the themes identified in this study, which are consistent with conclusions reported in similar qualitative research in this field (including studies on compression use for active VLUs). To address this, we have reviewed the manuscript and made minor revisions to the wording to ensure that the reporting reflects the exploratory and descriptive nature of our findings.
3- The use of multiple interview formats (e.g., Zooom, f2f) may have unintended effects on participant responses. This limitation should be clearly noted.	The decision to use multiple interview modalities was an intentional methodological choice aimed at maximising inclusion, accommodating participant preferences, and overcoming geographical and practical barriers to participation. While we recognise that the use of different data collection methods may have

	resulted in variations in the depth or nonverbal context of participant responses, this approach was prioritised to ensure equitable access and robust representation, in accordance with best practices for online and hybrid qualitative research. We have also acknowledged this as a limitation of the study and included it in the manuscript under the "strengths and limitations" section (Page 28).
4-Use fewer abbreviations. The journal's broad audience will better understand findings presented with full terminology in most cases.	Thank you for your valuable feedback. We have carefully reviewed the manuscript to incorporate the reviewer's comment. We made sure to define all abbreviations upon first use and maintained consistent usage throughout the text to enhance clarity. **No changes have been made to the transcript**
5-The COM-B framework terms (e.g., psychological capability, physical capability) in table 2 are nebulous and provide little insight into what is being assessed. Clarify these terms.	We appreciate the reviewer's valuable feedback. The COM-B framework and its links to the Theoretical Domains Framework (TDF) were already outlined in the ***Introduction*** to illustrate how they informed the study. To further enhance clarity, we have refined the title of an existing column in Table 2 to better reflect the description of how each COM-B component and corresponding TDF domain were applied in the context of this study. These revisions aim to make the theoretical constructs more transparent and accessible to readers.
6-Avoid using "first study (ex. P2, L57) " as this claim is difficult to substantiate and likely inaccurate.	Thank you for the comment! to our knowledge, based on a comprehensive literature search, no previous studies have specifically examined this topic using behaviour change theory. We have therefore revised the wording to be "*to our knowledge*, this is the first study to…" to convey this more appropriately.
7-All abbreviations or acronyms must be written in full on first use and kept consistent throughout. Current usage is repetitive and inconsistent.	We have thoroughly examined the manuscript to ensure that all abbreviations and acronyms are spelled out in full upon first use and are used consistently throughout. The required revisions have been made, and the changes are highlighted in blue.
8-Co-Occurring Diagnoses (P18, L31): Instead of listing general examples, provide specific co-occurring diagnoses for each individual to enhance relevance	We have revised the manuscript to specify the co-occurring diagnoses reported by participants and included the number of participants (*n)* for each condition (e.g., , arthriti.etc) (Page 18).

9-Many points in the Results section refer to data not cited in tables or figures, or at least no link to either. Ensure all references are properly referenced.	We appreciate the reviewer for pointing this out! We have reviewed the Results section and added explicit references to Table 2 wherever relevant.
10- If data are available, list the specific therapies used to treat ulcers. For example, participants undergoing hyperbaric oxygen therapy may have very different insights compared to those who did not undergo that type of treatment. The current data (Domain 4) are insufficient for interpretation without this context.	We appreciate the reviewer's suggestion to provide context for participant insights by including information about the specific ulcer treatments they received prior to the study. However, we did not collect therapy-specific data, as our study concentrated solely on the barriers and enablers to using post-healing prophylactic compression hosiery, rather than on factors related to active-phase ulcer treatments. All participants had previously healed venous leg ulcers, and the behavioural determinants identified—such as the social influences mentioned in Domain 4 (e.g., family support, stigma, community-based enablers like Leg Clubs)—are relevant at the prevention stage and are not dependent on the specific acute treatment used. We have clarified the study's inclusion criteria and focus on post-healing in the manuscript to enhance the contextual understanding. **No changes have been made to the manuscript**
11. Do not list individual researcher roles within the Methods and other sections. Summarize author roles collectively at the end of the manuscript.	We appreciate the reviewer's comment and have removed the author roles from the methods section, specifically regarding data collection and the Regiour and reflexivity section.
12. The study strengths and implications for practice are significantly overstated. The study is inherently interesting without generalizing the impact into findings that are ready to be used by other investigators. Reduce claims regarding broader impact and ensure any statement is consistent with the level of your data.	Thank you for the comment. The changes have been made in both sections and highlighted in blue.
Study Limitations: Expand this section to address issues noted in this review, including sample size, interview variability, lack of therapy-specific data, etc	The study limitations section has been expanded accordingly. However, regarding the lack of acute therapy-specific data, we have added clarification in our response above (point number 10)
Remove the future research section. The current study is quality-improvement oriented and not designed to establish a solid basis for future research.	We appreciate the reviewer's comment on the future research paragraph. This manuscript presents a theory-driven qualitative study grounded in implementation science, specifically utilising the COM-B model and the TDF.

	Implementation science involves a systematic process that begins with a diagnostic phase to identify the determinants of current practice, followed by a design phase in which these determinants guide targeted behaviour-change interventions. By mapping participants' barriers and enablers to the components of COM-B and the domains of the TDF, we have established a theory-based foundation that could be beneficial for developing, piloting on a small scale, and evaluating an intervention that addresses the identified determinants. This approach aligns with the UK Medical Research Council's guidance for complex interventions. Therefore, we suggest retaining a concise "Future Work" paragraph that outlines these next steps within the implementation science cycle. **NO changes have been made to the manuscript.**
I have serious concerns about the level and frequency of grammatical errors throughout. Significant revision is required to address these.	We appreciate the reviewer's feedback. We have carefully reviewed and revised the manuscript to address grammatical errors and enhance clarity. The revised version has also been reviewed by academic supervisors, who are native English speakers.

VERSION 2 - REVIEW

**Reviewer**	**2**
**Name**	**Garrison, Sheldon**
**Affiliation Institute**	**Advocate Aurora Health, Advocate Aurora Research**
**Date**	**19-Dec-2025**
**COI**	

Several comments remained unaddressed in the manuscript. It is important to address them before fully supporting this important work.

(Continued) Do not list individual researcher roles within the Methods and other sections. Summarize author roles collectively at the end of the manuscript.

Before the manuscript moves forward, the name of specific individuals doing the study needs to be removed before I can support this. Does the reader require that individual to repeat the study? While this question is rhetorical, it also highlights a baseline approach. Perhaps if a specific training is required (e.g., the study absolutely requires a specific physician subspecialist to do certain work then it is worthy of highlighting this. Otherwise, this needs to be removed. Similarly, there is no apparent benefit of highlighting the interviewer was a PhD candidate (non-clinician researcher would be sufficient). This should be revised throughout.

(Continued) Use fewer abbreviations. The journal's broad audience will better understand findings presented with full terminology in most cases. While I do appreciate the Author's ensuring that these have been described once, in it's current form the manuscript remains very difficult to follow outside of those who work in this field. The article may otherwise fit better within a more specialized journal.

(Continued) 8-Co-Occurring Diagnoses (P18, L31): Instead of listing general examples, provide specific co-occurring diagnoses for each individual to enhance relevance. I would recommend adding this to Table 2 or at least having a reference to a supplementary table for each individual. Please note individuals where co-occurring diagnoses were unable to be collected for the manuscript.

(Continued) Grammatical (e.g., there is no spacing with Table 2 (currently Table2) and the table itself has a mix between Centered and Left Justification. There remains many errors that would benefit from using the Word Editor and an approved large language model-based editor to ensure these are corrected for clarity.

VERSION 2 - AUTHOR RESPONSE

**Reviewer: 2**	**Author response and any edits made**<br>all the comments from the editor(s)' have been addressed and highlighted in Blue
Dr. Sheldon Garrison, Advocate Aurora Health<br>Comments to the Author:<br>Several comments remained unaddressed in the manuscript. It is important to address them before fully supporting this important work.<br><br>(Continued) Do not list individual researcher roles within the Methods and other sections. Summarize author roles collectively at the end of the manuscript.	Here are the points and how we addressed them:<br><br>(1) The text:'' During these visits, the researcher (BLINDED FOR REVIEW)…'', has been changed to:<br>**''…. During these visits, a member of the research team introduced'' (page 7).**

Before the manuscript moves forward, the name of specific individuals doing the study needs to be removed before I can support this. Does the reader require that individual to repeat the study? While this question is rhetorical, it also highlights a baseline approach. Perhaps if a specific training is required (e.g., the study absolutely requires a specific physician subspecialist to do certain work then it is worthy of highlighting this. Otherwise, this needs to be removed. Similarly, there is no apparent benefit of highlighting the interviewer was a PhD candidate (non-clinician researcher would be sufficient). This should be revised throughout	(2) **The text:''** Initial coding was carried out by two researchers: (***BLINDED FOR REVIEW,* female**), a PhD Candidate with an MSc in quality and safety in healthcare and (***BLINDED FOR REVIEW,* female*)***, PhD Candidate, with an MSc public heath, to ensure consistency and interpretive alignment.'' Names and qualifications have been removed and the text now is:<br><br>**''** Initial coding was carried out by two researchers to ensure consistency and interpretive alignment.'' **Page (10).**<br><br>**''** The wider research team, including (**Professor *BLINDED FOR REVIEW,* female*)***, an applied health researcher with expertise in wound care, and (**Professor *BLINDED FOR REVIEW, male***), an expert in health psychology and behaviour change, brought diverse disciplinary and experiential perspectives to the analysis'' has been changed to:<br><br>The wider research team, including **an applied health researcher with expertise in wound care** and **an expert in health psychology and behaviour change**, brought diverse disciplinary and experiential perspectives to the analysis 2. These varied perspectives enabled a critical examination of underlying assumptions, challenged conventional interpretations, and deepened analytic insights'' **(Page 10).**<br><br>**We suggest retaining the description of the research team's expertise in wound care and health psychology, as it was vital to the credibility of the analysis. Describing these complementary areas of expertise demonstrates reflexivity, aligns with reporting standards (e.g., COREQ), and strengthens rigour.**
(Continued) Use fewer abbreviations. The journal's broad audience will better understand findings presented with full terminology in most cases. While I do appreciate the Author's ensuring that these have been described once, in it's current form the manuscript remains very difficult to follow outside of those who work in this field. The article may otherwise fit better	We appreciate the reviewer's concern regarding accessibility for a broad audience.<br>**Action Taken:** We have undertaken a thorough revision to enhance clarity and readability for general audience.<br><br>- We reduced the use of abbreviations throughout the manuscript and used the complete terminology. The following

within a more specialized journal.	abbreviations have been removed and replaced with full terminology.<br>These are:<br>**Behaviour Change Wheel (BCW)** was removed from the abstract (Page 1), Introduction (Page 6), Discussion (24).<br>**Intervention Functions (Ifs):** was removed from the abstract (Page 2), Introduction (Page 6), and in Table 2.<br>**Behaviour Change Techniques (BCTs):** was removed from the abstract (Page 2), Introduction (Page 6), and in Table 2.<br><br>- Four abbreviations have been retained: venous leg ulcers (VLUs), the Capabilities, Opportunities and Motivations model of Behaviour change (COM-B), the Theoretical Domains Framework (TDF), and NHS. These terms are established conceptual labels within the literature. Their repeated use reflects their central role in the study. Continued use of the full terms would disrupt narrative flow and obscure conceptual linkages across sections.<br><br>We confirm that the retained abbreviations are defined at first mention, used consistently throughout the text.
(Continued) 8-Co-Occurring Diagnoses (P18, L31): Instead of listing general examples, provide specific co-occurring diagnoses for each individual to enhance relevance. I would recommend adding this to Table 2 or at least having a reference to a supplementary table for each individual. Please note individuals where co-occurring diagnoses were unable to be collected for the manuscript.	**Action Taken:**<br>We have added **Supplementary File E**, which presents participant-level co-occurring diagnoses A corresponding sentence has been inserted and highlighted in the manuscript (**page 11)** to direct readers to this supplementary material.
Continued) Grammatical (e.g., there is no spacing with Table 2 (currently Table2) and the table itself has a mix between Centered and Left Justification. There remains many errors that would benefit from using the Word Editor and an approved large language model-based editor to ensure these are corrected for clarity.	Thank you for this comment. All grammatical, spacing, and formatting issues, in Table 2, have been corrected
